# Associations between the performance of vertical jump and accelerative sprint in elite sprinters

**DOI:** 10.3389/fbioe.2025.1539197

**Published:** 2025-04-29

**Authors:** Junliang He, Ming Li, Qiuping Zhang, Zhiye Zhang

**Affiliations:** ^1^ Shanghai Research Institute of Sports Science, Shanghai Anti-Doping Agency, Shanghai, China; ^2^ Kunwei Sport Technology (Shanghai) Co., Ltd., Shanghai, China

**Keywords:** countermovement jump, squat jump, sprint kinetics, sprint, sports performance

## Abstract

**Objective:**

The purpose of this study is to investigate the relationship between components of the Sprint Profile during acceleration and kinematic and kinetic measures of the Counter Movement Jump (CMJ) and Squat Jump (SJ), to determine whether jump performance can monitor acceleration performance in sprinting.

**Methods:**

Eight elite sprinters offered to participate in the study (mean ± SD: age 21.43 ± 3.6 years; height 171.58 ± 7.76 cm; weight 54.71 ± 6.05 kg). The training age of athletes was 8.86 ± 4.30 years, which included SJ, CMJ, and accelerative sprint tests.

**Results:**

Significant negative correlations were found between propulsion time and braking time during sprint acceleration and CMJ metrics, including flight time, jump height, vertical take-off velocity, and push impulse (r = −0.598 to −0.721, *p* < 0.01). Similar associations were observed for SJ variables, though generally with slightly lower correlation strength. Ground contact time during sprinting was positively correlated with CMJ and SJ metrics (*p* < 0.05). Additionally, several sprint-phase kinetic variables—such as horizontal and vertical propulsion impulses—showed significant negative correlations with both CMJ and SJ outcomes. These findings suggest that specific jump performance measures, particularly from CMJ, may serve as effective monitor of acceleration sprint performance.

**Conclusion:**

This study confirms that key countermovement jump and squat jump metrics, especially jump height and flight time, are significantly associated with sprint acceleration in elite athletes. These findings support the use of jump tests as practical tools to monitor and enhance acceleration performance through targeted lower-limb power training.

## 1 Introduction

Sprinting is crucial in many sports activities, requiring athletes to cover a specific distance rapidly by attaining maximal speed and demonstrating substantial forward acceleration. This ability is related to generating and applying a large amount of power output in the horizontal direction on the ground, which means a large amount of horizontal external force that exceeds the sprint acceleration at different speeds (Morin et al., 2011b, 2011a; Rabita et al., 2015; Colyer et al., 2018; Sugisaki et al., 2025). Additionally, elite sprinters require not only exceptional speed but also significant strength qualities. Consequently, their training typically involves high-intensity sprint exercises, generally performed at intensities ranging from 80% to 100% of their maximum sprinting capacity (Haugen et al., 2019).

In previous studies, sprinting over distances and jumping are routinely used by coaches to assess the athletic performance of athletes (Sado et al., 2023). However, the correlation of these necessitates still remains to be studied. Previous studies have established general correlations between jump performance and sprint completion time or speed across different sports (Loturco et al., 2015; Furlong et al., 2021). However, it remains unclear whether improvements in jump performance directly correspond to specific gait patterns or movement strategies during the acceleration phase of sprinting. Additionally, there is a lack of systematic research investigating the potential of these jump metrics to monitor kinematic performance during sprint acceleration. Exploring the correlation between jump metrics and specific kinematic parameters during the sprint acceleration phase will provide deeper insights into the underlying mechanisms of performance enhancement and offer more precise guidance for athlete-specific training.

It is widely known that force production, the rate of force development, and the effective application of force are vital to utmost importance for sprinting (Hicks et al., 2020). Therefore, during the execution of Counter Movement Jump (CMJ) and Squat Jump (SJ), the rapid generation of forces is indispensable for athletes to propel their center of mass upward or forward during jumping (Keiner et al., 2022; Barrera et al., 2023). CMJ refers to a vertical jump performed with an initial downward movement (countermovement) before the upward thrust, while SJ denotes a vertical jump executed from a static squat position without any preparatory countermovement. Previous research has indicated that horizontal force production plays a critical role during the sprint acceleration phase, while its importance diminishes in the maximal velocity phase, where the emphasis shifts to vertical force generation (Hicks et al., 2020). Furthermore, most of the current literature primarily focuses on the relationship between competition results and the first 30–40 m of sprinting (namely, the sprint acceleration phase). Nevertheless, there is a lack of in-depth exploration of the relationship between dynamics and jumping parameters during an athlete’s accelerated running.

Therefore, the purposes of this study were 1) to examine the relationship between components of the Sprint Profile during acceleration and various kinematic and kinetic measures of the CMJ and SJ and 2) to ascertain the monitor capability of jump performance measures during the stage of acceleration sprint. We hypothesized that there would be significant correlations between jump performance variables and the single-ground contact kinetics observed during the sprint acceleration phase.

## 2 Materials and methods

### 2.1 Participants

Eight elite sprinters participated in the study (mean ± SD: age 21.43 ± 3.60 years; height 171.58 ± 7.76 cm; weight 54.71 ± 6.05 kg; training years 8.86 ± 4.30 years). All participants were elite sprinters at least. They had no history of upper and lower extremity diseases or deformities. Moreover, they had remained uninjured for at least 6 months prior to the test. All participants were athletes with training experience. Four weeks before the measurement was conducted, they had become thoroughly familiar with all test procedures. This experimental procedure was approved by the local ethics committee. After the purposes, benefits, and potential risks involved were explained, all athletes provided written informed consent for the research. This study has been approved by the Ethics Committee of the Shanghai Institute of Sports Science (Approval No. LLSC20230011). Informed consent was obtained from all participants prior to the commencement of the study. All subjects were tested during the preparation period (from November to March).

### 2.2 Testing procedures

#### 2.2.1 Sprint session

**FIGURE 1:**
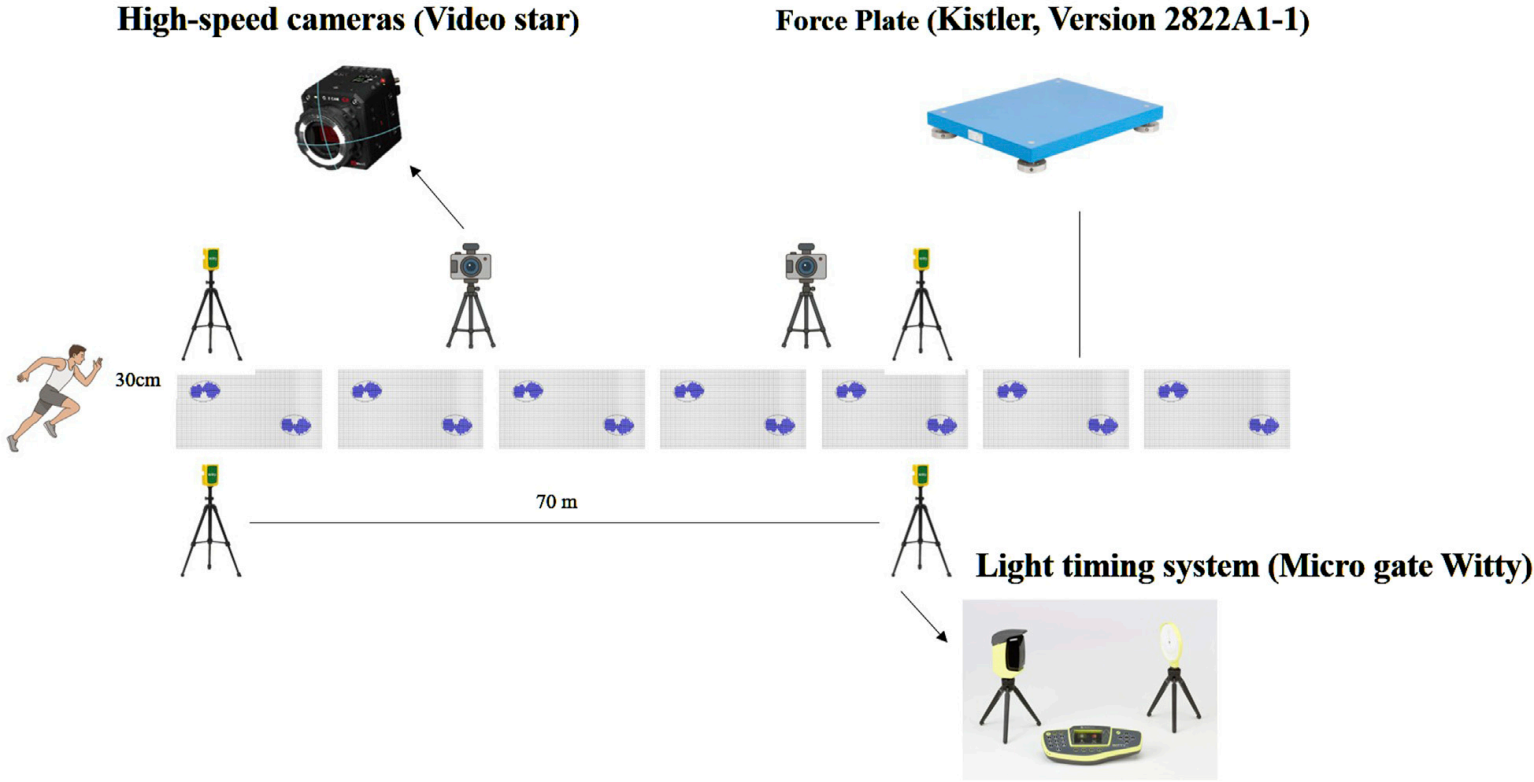
Test site equipment layout.

The testing procedures for this study were conducted in a professional indoor athletic stadium (see Figure 1), utilizing 2 force measuring tables (Kistler, Version 2822A1-1, Winterthur, Switzerland) and 2 high-speed cameras (Video star Co, Ltd, Z Cam E2, Beijing, China). The force measuring tables had a sampling frequency of 1,000 Hz while the camera sampling frequency was set at 200 Hz. Prior to the sprints, each subject completed a standardized warm-up supervised by their coach. Then, athletes are required to start from the starting line with a standing starts and perform two to three accelerated sprints. The accelerative sprint was measured using the light timing system (Micro gate Witty, America), which consisted of two pairs of light gates placed at 0 and 20 m with a height of 70 cm. Participants started from a 2-point stance 30 cm behind the starting line to avoid triggering the timing gates before the test began (Ye et al., 2024). Data of a single ground contact were collected during the acceleration sprint process, and participants performed more than three repetitions if they thought that maximum effort could be improved by conducting the test again. To ensure consistency, an experienced starter provided standard starting commands to the athletes, including a verbal cue of “on your mark” was given, followed by a verbal cue of “set.” Once the participants were in the set position, an electronic starting gun, which also functioned as the trigger for data collection, was employed to initiate the trials (see Figure 1). There was a minimum of 3 min recovery between sprints. An extended force platform system consisting of 2 force platforms covered in synthetic material and connected to a single computer (DELL, Precision T7920, USA) was used to measure GRFs during sprinting from approximately 14.7 m behind the starting line to the 17.1 m mark. The force platforms covered a distance from 14.7 m to 17.1 m. A 3-minute rest was taken between each test to ensure full recovery. Athletes performed sprints wearing their own training clothing and track spike shoes. The fastest sprint time was retained for statistical analysis.

#### 2.2.2 Jump session

**FIGURE 2:**
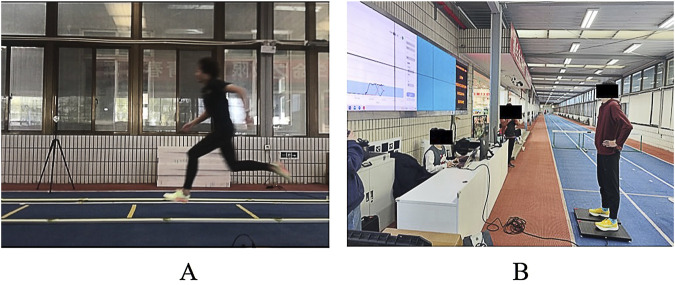
**(A)** Accelerated running test; **(B)** jumping test.

Prior to the jump assessments, athletes performed standardized warm-up. Two types of jump assessments, the SJ and CMJ, were performed by each athlete, both of which are extensively used in literature (Stavridis et al., 2019). Each jump assessment was conducted in a randomized order. For each jump assessment, three trials were performed. All vertical jumps were carried out bilaterally (Figure 2).

**FIGURE 3:**
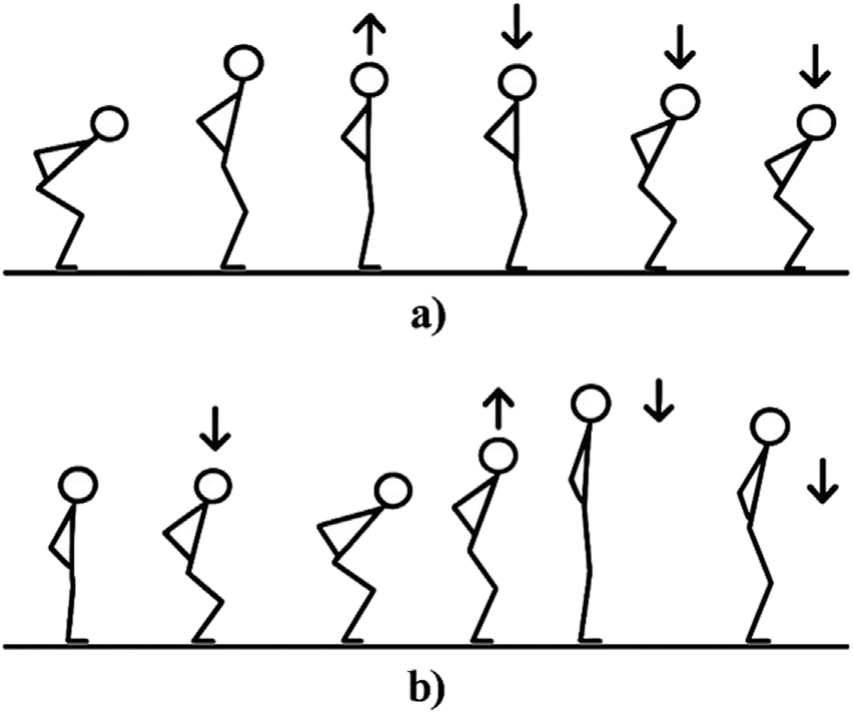
Vertical jump assessments performed (Maulder et al., 2006). **(a)** Squat jump sequence. **(b)** Countermovement jump sequence.

During the squat jump assessment, athletes were instructed to maintain a knee-bent position (with a knee angle of approximately 120°) for 3 seconds while keeping their hands on their hips (Figure 3a). Upon an auditory cue after 3 seconds, athletes executed a maximal vertical jump without performing any countermovement or downward motion prior to take-off. A successful trial was confirmed by observing no visible sinking or countermovement before the jump execution.

The movement requirements for the CMJ were that athletes started with their hands on their hips, determined the degree of knee flexion by themselves, and then jumped upward as high as possible in the subsequent concentric phase (see Figure 3b).

Before conducting the formal lower limb explosive power test, each subject performed 2-3 practices. There was an interval of 30–60 s between each jump to ensure full recovery. Athletes performed jumps in their usual training clothes and running shoes. The test with the best athletic performance of each athlete was selected and used for further analysis.

### 2.3 Signal processing

Kinetic data collected from the force platforms were processed using a fourth-order Butterworth low-pass digital filter with a cut-off frequency of 14 Hz, as recommended by previous research (Keiner et al., 2022; Barrera et al., 2023). Kinematic data from high-speed cameras were analyzed using Video star to extract variables such as joint angles, velocities, and accelerations. All raw signals were visually inspected for accuracy, and any anomalies or data artifacts were removed prior to statistical analyses. Data synchronization between kinetic and kinematic measurements was ensured by aligning the timestamps from the force platforms and the video recordings.

### 2.4 Statistical analysis

The normality of all variables was assessed using the Shapiro–Wilk test. Data are presented as mean ± standard deviation. Pearson correlation analyses were conducted to examine relationships among sprint, SJ, and CMJ performance variables using SPSS Statistics software (IBM SPSS Statistics 25, Armonk, NY, USA). The qualitative interpretation of correlation coefficients was as follows: <0.09, trivial; 0.10–0.29, small; 0.30–0.49, moderate; 0.5–0.69, large; 0.70–0.89, very large; >0.90 nearly perfect (Arteaga et al., 2000). Negative correlation coefficients indicated inverse relationships between variables. Statistical significance was set at *p* < 0.05, and a more stringent significance level was established at *p* < 0.01.

## 3 Results

The mean acceleration sprint time (As) was 2.87 ± 0.20 s. Key kinetic variables during the sprint phase included Bt (0.01 ± 0.002 s), Pt (0.11 ± 0.01 s), Ct (0.12 ± 0.01 s), and Fmax (1842.68 ± 218.02 N). The impulses during braking and propulsion phases, including HI_1_, VI_1_, CI_1_, HI_2_, VI_2_, and CI_2_, demonstrated considerable variability across participants. For CMJ, the mean H was 0.41 ± 0.08 m, Ft was 0.574 ± 0.05 s, V_1_ was 2.82 ± 0.29 m/s, and PI was 296,118.35 ± 43,672.17 n·s. The peak force (F_0_) during CMJ was 1,337.84 ± 122.34 N. For SJ, the results were similar, with H1 of 0.40 ± 0.08 m, Ft_1_ of 0.57 ± 0.05 s, V_2_ of 2.79 ± 0.26 m/s, and PI_1_ of 285,794.07 ± 38,394.05 n·s. Peak force (F_3_) during SJ was 1,338.13 ± 113.10 N. These results provide the baseline characteristics for subsequent correlation analyses (Table 1).

**TABLE 1:** Means ± standard deviations, sprint performance, sprint measures and jump performance measures.

Parameters	Mean ± SD
Sprint performance measures	
As(s)	2.87 ± 0.20
Bt(s)	0.01 ± 0.002
Pt(s)	0.11 ± 0.01
Ct (s)	0.12 ± 0.01
Fmax(N)	1842.68 ± 218.02
F_1_(N)	732.02 ± 685.96
HI_1_(n·s)	10.24 ± 11.94
VI_1_(n·s)	29.31 ± 25.00
CI_1_(n·s)	6.08 ± 6.98
F_2_(N)	50,394.36 ± 4,847.80
HI_2_(n·s)	2,351.90 ± 408.10
VI_2_(n·s)	14,574.29 ± 2091.99
CI_2_(n·s)	5,749.59 ± 692.10
Countermovement Jump measures	
H (m)	0.41 ± 0.08
F_0_ (N)	1,337.84 ± 122.34
Ft (s)	0.574 ± 0.05
V_1_ (m/s)	2.82 ± 0.29
PI (n·s)	296,118.35 ± 43,672.17
Squat Jump measures	
H_1_ (m)	0.40 ± 0.08
F_3_ (N)	1,338.13 ± 113.10
Ft_1_ (s)	0.57 ± 0.05
V_2_ (m/s)	2.79 ± 0.26
PI_1_ (n·s)	285,794.07 ± 38,394.05

As, accelerative sprint; Bt, braking time; Pt, propulsion time; Ct, contact time; Fmax, maximal theoretical force; F_1_, The resultant force of braking phase; HI_1_, impulse of horizontal braking; VI_1_, impulse of vertical braking; CI_1_, impulse of combined braking; F_2_, The resultant force of pushing phase; HI_2_, impulse of horizontal propulsion; VI_2_, impulse of vertical propulsion; CI_2_, impulse of combined propulsion; H, height (m); CMJ: F_0_: Peak force (N); Ft, flight time (s); V_1_, vertical velocity at take-off (m/s); PI, Push impulse (n·s). SJ: F_3_: Peak force (N); Ft_1_, flight time (s); V_2_, vertical velocity at take-off (m/s); PI_1_, Push impulse (n·s).

The Bt of sprint performance was negatively associated with Ft (p < 0.01, r = −0.654), H (*p* < 0.01, r = −0.658), V_1_ (*p* < 0.01, r = −0.654), and PI (*p* < 0.01, r = −0.680) of CMJ. The Pt of sprint performance was negatively associated with Ft (*p* < 0.01, r = −0.598), H (*p* < 0.01, r = −0.603), V_1_ (*p* < 0.01, r = −0.598), and PI (*p* < 0.01, r = −0.721) of CMJ. The Ct of sprint performance was positively associated with Ft (*p* < 0.05, r = 0.581), H (*p* < 0.05, r = 0.591), V_1_ (*p* < 0.05, r = 0.581), and PI (*p* < 0.01, r = 00.576) of CMJ. The F_1_ of sprint performance was negatively associated with Ft (*p* < 0.05, r = 0.586), H (*p* < 0.01, r = −0.592), and V_1_ (p < 0.05, r = −0.586) of CMJ. The F_2_ of sprint performance was negatively associated with H (*p* < 0.05, r = 0.471) of CMJ. The HI_2_ of sprint performance was negatively associated with Ft (p < 0.05, r = −0.580), H (*p* < 0.05, r = −0.568), and V_1_ (*p* < 0.05, r = −0.580) of CMJ. The VI_2_ of sprint performance was negatively associated with Ft (*p* < 0.01, r = −0.592), H (*p* < 0.05, r = −0.581), and V_1_ (*p* < 0.01, r = −0.592) of CMJ (Table 2).

**TABLE 2:** Pearson correlation analysis between Sprint performance measures and Countermovement Jump measures.

Parameters	F_0_ (N)	Ft (s)	H (m)	V_1_ (m/s)	PI (n·s)
As(s)	−0.138	0.265	0.260	0.265	−0.171
Bt(s)	0.061	−0.654**	−0.658**	−0.654**	−0.680**
Pt(s)	0.059	−0.598**	−0.603**	−0.598**	−0.721**
Ct (s)	0.181	0.581*	0.591*	0.581*	0.576**
Fmax(N)	0.439	−0.021	−0.020	−0.021	0.228
F_1_(N)	−0.363	−0.586*	−0.592**	−0.586*	−0.446
HI_1_(n·s)	0.457	0.246	0.247	0.246	0.258
VI_1_(n·s)	0.372	−0.052	−0.053	−0.052	0.129
CI_1_(n·s)	0.461	−0.140	−0.123	−0.140	0.447
F_2_(N)	0.244	0.466	0.471*	0.466	0.344
HI_2_(n·s)	−0.053	−0.580*	−0.568*	−0.580*	−0.115
VI_2_(n·s)	0.064	−0.592**	−0.581*	−0.592**	−0.134
CI_2_(n·s)	−0.138	0.265	0.260	0.265	−0.171

**p < 0.01; *p < 0.05.

As, accelerative sprint; Bt, braking time; Pt, propulsion time; Ct, contact time; Fmax, maximal theoretical force; F_1_, The resultant force of braking phase; HI_1_, impulse of horizontal braking; VI_1_, impulse of vertical braking; CI_1_, impulse of combined braking; F_2_, The resultant force of pushing phase; HI_2_, impulse of horizontal propulsion; VI_2_, impulse of vertical propulsion; CI_2_, impulse of combined propulsion; H, height (m); CMJ: F_0_: Peak force (N); Ft, flight time (s); V_1_, vertical velocity at take-off (m/s); PI, Push impulse (n·s).

The Bt of sprint performance was negatively associated with Ft_1_ (*p* < 0.01, r = −0.679), H_1_ (*p* < 0.01, r = −0.679), V_2_ (*p* < 0.01, r = −0.679), and PI_1_ (*p* < 0.05, r = −0.582) of SJ. The Pt of sprint performance was negatively associated with Ft_1_ (*p* < 0.01, r = −0.625), H_1_ (*p* < 0.01, r = −0.628), V_2_ (*p* < 0.01, r = −0.625), and PI_1_ (*p* < 0.01, r = −0.628) of SJ. The Ct of sprint performance was positively associated with Ft_1_ (*p* < 0.05, r = 0.565), H_1_ (*p* < 0.05, r = 0.580), V_2_ (*p* < 0.05, r = 0.565), and PI_1_ (*p* < 0.05, r = 0.573) of SJ. The F_1_ of sprint performance was negatively associated with Ft_1_ (*p* < 0.05, r = −0.588), H_1_ (*p* < 0.01, r = −0.590), and V_2_ (*p* < 0.05, r = −0.588) of SJ. The F_2_ of sprint performance was positively associated with Ft_1_ (*p* < 0.05, r = 0.498), H_1_ (*p* < 0.05, r = 0.506), and V_2_ (*p* < 0.05, r = 0.498) of SJ. The HI_2_ of sprint performance was negatively associated with Ft_1_ (*p* < 0.05, r = −0.543), H_1_ (*p* < 0.05, r = −0.526), and V_2_ (*p* < 0.05, r = −0.543) of SJ. The VI_2_ of sprint performance was negatively associated with Ft_1_ (*p* < 0.05, r = −0.544), H_1_ (*p* < 0.05, r = −0.528), and V_2_ (*p* < 0.05, r = −0.544) of SJ. The CI_2_ of sprint performance was negatively associated with Ft_1_ (*p* < 0.05, r = −0.544), H_1_ (*p* < 0.05, r = −0.528), and V_2_ (*p* < 0.05, r = −0.544) of SJ (Table 3).

**TABLE 3:** Pearson correlation analysis between Sprint performance measures and Squat Jump measures.

Parameters	F_3_ (N)	Ft_1_ (s)	H_1_ (m)	V_2_ (m/s)	PI_1_ (n·s)
As(s)	0.263	0.267	0.254	0.267	−0.245
Bt(s)	−0.419	−0.679**	−0.679**	−0.679**	−0.582*
Pt(s)	−0.362	−0.625**	−0.628**	−0.625**	−0.628**
Ct (s)	0.261	0.565*	0.580*	0.565*	0.573*
Fmax(N)	0.330	0.000	0.000	0.000	0.445
F_1_(N)	−0.398	−0.588*	−0.590**	−0.588*	−0.341
HI_1_(n·s)	0.441	0.249	0.247	0.249	0.418
VI_1_(n·s)	0.298	−0.040	−0.042	−0.040	0.364
CI_1_(n·s)	0.463	−0.056	−0.036	−0.056	0.738**
F_2_(N)	0.312	0.498*	0.506*	0.498*	0.414
HI_2_(n·s)	0.060	−0.543*	−0.526*	−0.543*	0.165
VI_2_(n·s)	0.059	−0.544*	−0.528*	−0.544*	0.166
CI_2_(n·s)	0.059	−0.544*	−0.528*	−0.544*	0.166

** p < 0.01; *p < 0.05

As, accelerative sprint; Bt, braking time; Pt, propulsion time; Ct, contact time; Fmax, maximal theoretical force; F_1_, The resultant force of braking phase; HI_1_, impulse of horizontal braking; VI_1_, impulse of vertical braking; CI_1_, impulse of combined braking; F_2_, The resultant force of pushing phase; HI_2_, impulse of horizontal propulsion; VI_2_, impulse of vertical propulsion; CI_2_, impulse of combined propulsion; H, height (m); SJ: F_3_: Peak force (N); Ft_1_, flight time (s); V_2_, vertical velocity at take-off (m/s); PI_1_, Push impulse (n·s).

## 4 Discussion

The primary findings of this study demonstrated significant correlations between sprint acceleration performance and jump performance metrics derived from both CMJ and SJ. Specifically, shorter Bt and Pt during sprinting were strongly associated with superior jump performance, longer Ct was associated with reduced jump performance. Notably, CMJ metrics, particularly PI, demonstrated slightly stronger correlations than SJ metrics with key sprint parameters such as Pt and Bt.

### 4.1 Force-velocity relationship in sprint acceleration

A great number of previous studies have delved into the relationship between strength and sprinting. However, there is a scarcity of research focusing on the correlation between force and the single-ground contact dynamics during the sprint acceleration. Previous studies have shown that the ability to produce high levels of horizontal power during a sprint is significantly associated with sprint performance (Morin et al., 2012; Cross et al., 2015; Delaney, 2018) which is consistent with our research. While certain studies have claimed significant correlations between force and accelerative sprint (Marques et al., 2011). This discrepancy may be attributed to the intricate nature of accelerative sprinting, which necessitates coordinated muscle actions across multiple joints. Nonetheless, our analysis revealed a positive correlation between maximal theoretical force and performance in both countermovement jump and squat jump among elite sprinters. This finding is consistent with previous research suggesting that higher maximal theoretical force contributes to faster acceleration in both sprinters and hurdlers (Rabita et al., 2015; Jiménez-Reyes et al., 2022). Therefore, it is indicated that CMJ and SJ may be used as effective means to evaluate the maximal theoretical force of elite sprinters in the accelerated sprinting stage.

Previous research has reported a significant negative correlation (*p* < 0.05) between Ct and accelerative sprint (Morin et al., 2012). This finding is inconsistent with our results. Meanwhile, Brughelli et al. reported that rugby players increased their stride rate and stride flight and reduced their Ct at faster speeds (Brughelli et al., 2011). In our study, ground Ct showed positive correlations with CMJ Ft, H, and V_1_. This suggested that longer Ct might enable more complete triple extension during CMJ execution, thereby improving jump performance. However, previous studies consistently demonstrated that shorter Ct enhances acceleration sprint performance (Hayes and Caplan, 2012). Therefore, based on our findings, we recommend that participants in this study implement training protocols specifically targeting Ct reduction, such as plyometric exercises emphasizing rapid stretch-shortening cycles (SSC) (Markovic and Mikulic, 2010). Our study demonstrated that the flight time, flight height, velocity at take-off, and performance index of CMJ were significantly and negatively correlated with both the Pt of accelerative sprint (*p* < 0.01). Meanwhile, a statistically significant positive correlation was observed with acceleration sprint Ct (*p* < 0.05). In summary, the present study revealed that ground Ct was positively correlated with jumping performance, which contrasted with previous findings demonstrating negative correlations with sprint acceleration performance. Based on these results, we recommended plyometric training was suggested to reduce Ct for acceleration-dependent disciplines, while maintaining appropriate Ct might optimize power output for jumping events.

### 4.2 Correlations between CMJ and SJ kinematic parameters and sprint acceleration performance

Our results demonstrated that flight time, flight height, take-off velocity, and push impulse during both CMJ and SJ were significantly negatively correlated with Pt, Bt, and Ct during accelerative sprinting (*p* < 0.01; *p* < 0.05). However, contact time is an indicator to measure the performance of accelerated running (Morin et al., 2012). This further proves the correlation between CMJ or SJ and accelerative sprint. Furthermore, the flight height demonstrated a significant negative correlation with accelerative sprint performance. Previous research in male rugby players has reported that both the CMJ height and the SJ height were significantly associated with the completion time of acceleration sprint 30 and the height of CMJ (r = −0.66) in rugby union players (Jiménez-Reyes et al., 2018). Similarly, elite sprinters have been found to have a strong correlation between the CMJ height (r = 0.84) and acceleration sprint speed (Loturco et al., 2015). Our study revealed a significant negative correlation between the CMJ and SJ height of elite sprinters and the performance of accelerative running. Some studies have already indicated that in male athletes, both vertical and horizontal jumping abilities are related to sprint performance (Washif and Kok, 2022). In line with our results, previous studies have documented a statistically significant inverse relationship between CMJ jump height and sprint velocity production in the initial acceleration phase (Washif and Kok, 2022; Barrera et al., 2023). Therefore, it was suggested that the CMJ and SJ height is a significant metric to consider when developing accelerative sprint performance in elite sprinters.

### 4.3 Correlations between CMJ and SJ kinetic parameters and sprint acceleration performance

The vertical impulse is considered a significant determinant of sprinting ability. However, Little and Williams (2005) investigated the vertical impulse of a concentric squat jump was nonsignificant correlated to accelerative sprint. Our research contradicts this finding, which may be attributed to the difference in running style between elite sprinters and sprinters. However, in our study, we found that the horizontal braking impulse during the braking phase has a significant correlation with CMJ and SJ performance. The application of horizontal force seems to be crucial for acceleration sprinting (Dietze-Hermosa et al., 2021). The current results corroborated these findings, demonstrating significantly stronger correlations of horizontal force with jump height than vertical take-off velocity or flight time during both CMJ and SJ performances. Our results similarly demonstrated that horizontal force application remained critical during the sprint acceleration phase. Likewise, current literature indicates that the application of horizontal force is a crucial factor in determining sprint speed for high-level sprinters. Additionally, increased horizontal force results in higher maximal sprint velocities and ultimately leads to a greater horizontal impulse (Samozino et al., 2016; Hicks et al., 2020). Therefore, elite sprinters attaining higher maximal sprint velocities through HI_1_ application may be able to gain advantages in the final sprint process of the competition. Both countermovement jumps and squat jump showed significant correlations with sprint acceleration performance metrics, indicating their potential to monitor sprint acceleration. However, countermovement jumps metrics, particularly push impulse, demonstrated slightly stronger correlations than squat jump metrics with key sprint parameters such as propulsion time and braking time.

### 4.4 Study limitations

This study has several limitations that should be acknowledged. First, although elite sprinters were recruited, the small sample size (n = 8) may limit the generalizability of the findings. A longitudinal design would be more appropriate to provide a comprehensive understanding of these relationships over time. Additionally, detailed kinematic analyses of the sprint acceleration phase were not conducted. Future studies should explore the effects of different training interventions on acceleration performance and aim to identify reliable monitor indicators to guide performance enhancement in elite sprinters.

### 4.5 Applications

In training practice, coaches should consider CMJ and SJ as monitoring indicators for middle -distance runners to evaluate the acceleration ability of athletes during competition. Specifically, tracking CMJ parameters such as jump height, flight time, and vertical take-off velocity is essential, as improvements in these measures may help reduce ground contact time and enhance horizontal braking impulse during the braking phase as well as maximal theoretical force during sprinting. Developing these parameters may be associated with an improved ability to generate higher speeds during critical moments of a race.

## 5 Conclusion

This study confirms that key CMJ and SJ metrics, particularly jump height and flight time, are significantly related to sprint acceleration performance in elite athletes. These findings highlight the value of jump-based assessments in monitoring acceleration capability. Incorporating lower-limb explosive strength training and using CMJ or SJ as practical monitoring tools may enhance sprint-specific performance, especially during the acceleration phase.

## Data Availability

The original contributions presented in the study are included in the article/Supplementary Material, further inquiries can be directed to the corresponding author.
