# Supplementary figures and images for: Associations between the performance of vertical jump and accelerative sprint in elite sprinters
Source: Front Bioeng Biotechnol. 2025 Apr 29;13:1539197. doi: 10.3389/fbioe.2025.1539197 (PMC12070001; doi:10.3389/fbioe.2025.1539197)

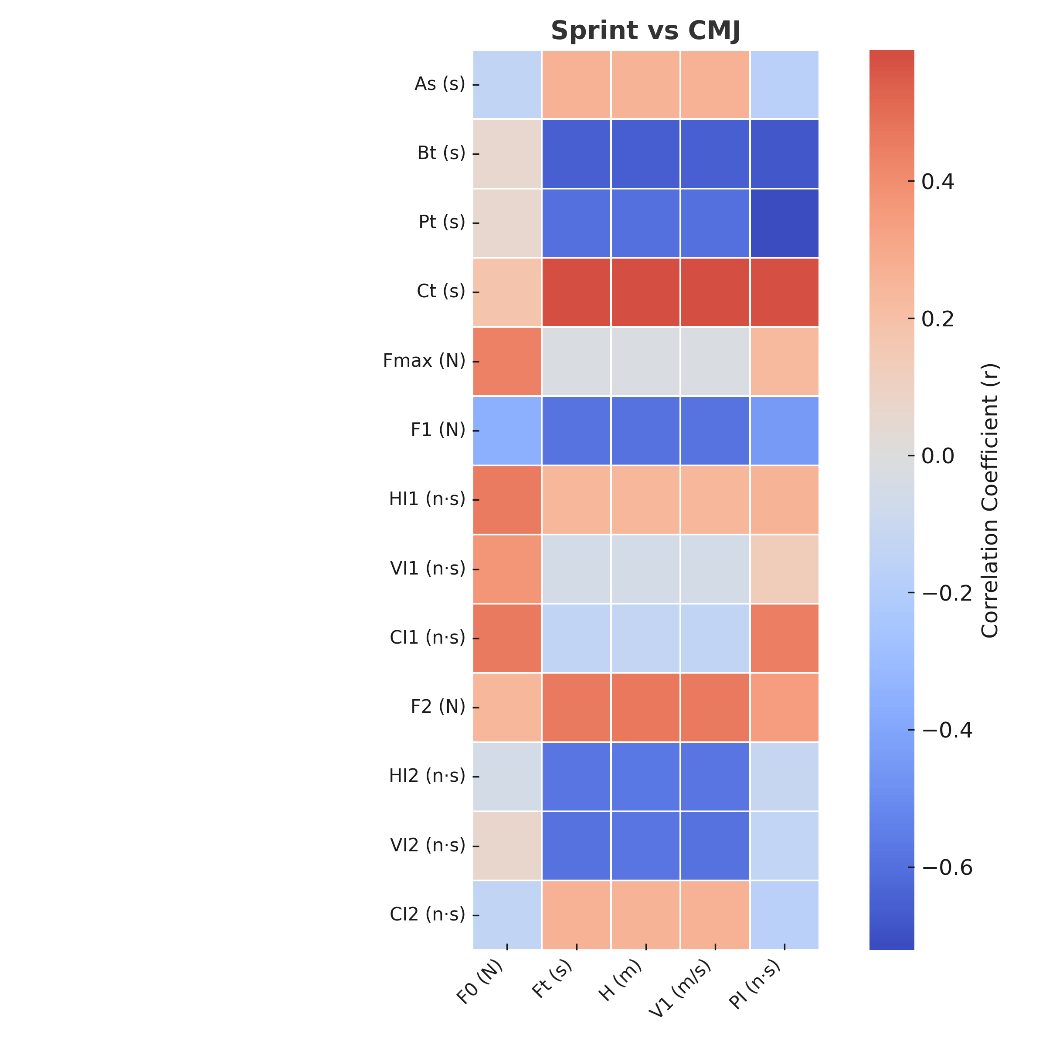

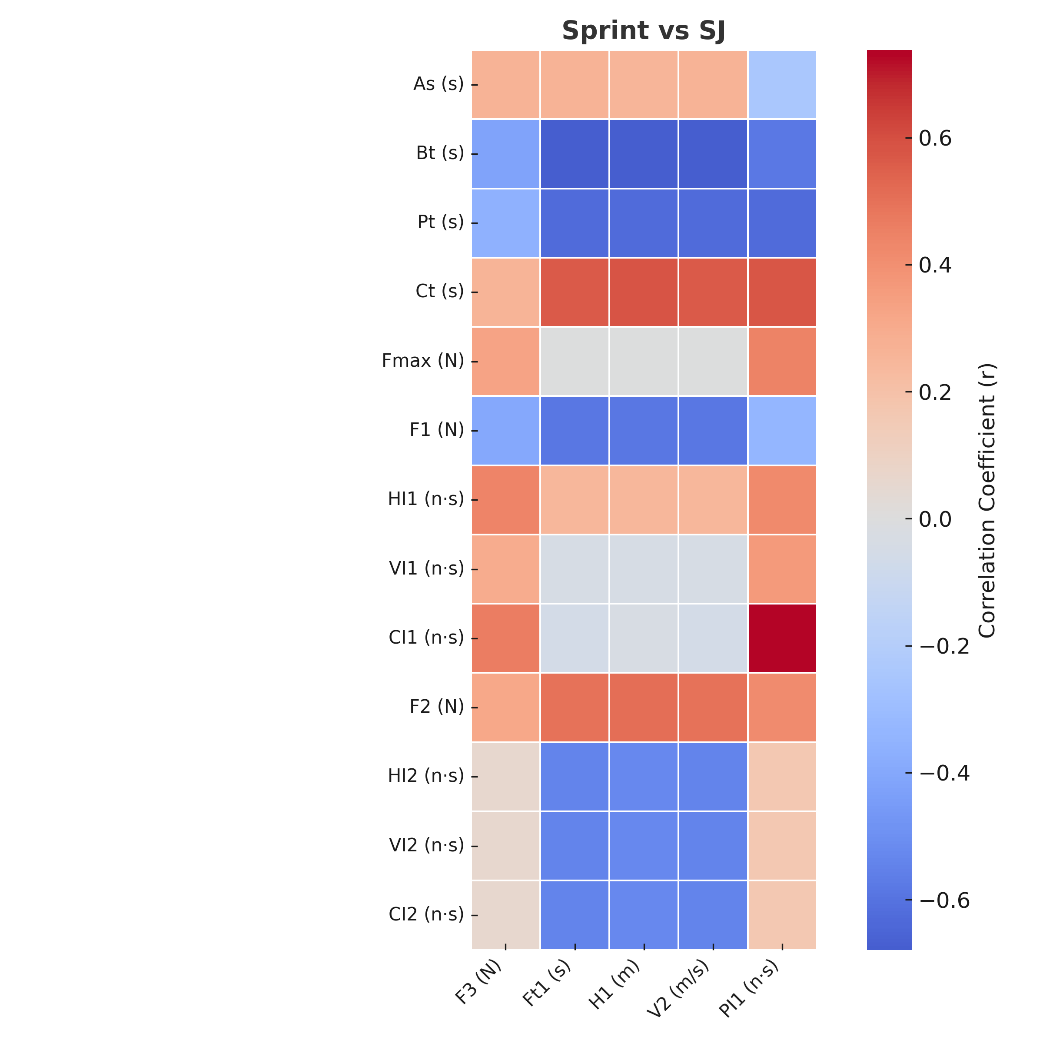
Figure 1. Heatmap of Pearson correlations between sprint performance and SJ and CMJ metrics.

Supplement: Supplementary file 1 [file Table1.docx]
